# Effects of Probiotics on Metabolic Syndrome: A Systematic Review of Randomized Clinical Trials

**DOI:** 10.3390/nu12010124

**Published:** 2020-01-01

**Authors:** Carmen Tenorio-Jiménez, María José Martínez-Ramírez, Ángel Gil, Carolina Gómez-Llorente

**Affiliations:** 1 Endocrinology and Nutrition Clinical Management Unit, University Hospital Virgen de las Nieves, 18014 Granada, Spain; 2 Endocrinology and Nutrition Clinical Management Unit, University Hospital of Jaén, 23007 Jaén, Spain; mjmartin@ujaen.com; 3 Department of Health Sciences, School of Health Sciences, University of Jaén, 23071 Jaén, Spain; 4 Department of Biochemistry and Molecular Biology II, School of Pharmacy, University of Granada, 18071 Granada, Spain; agil@ugr.es; 5 Institute of Nutrition and Food Technology “José Mataix”, Center of Biomedical Research, University of Granada, 18016 Granada, Spain; 6 Instituto de Investigación Biosanitaria ibs. GRANADA, 18012 Granada, Spain; 7 CIBEROBN (CIBER Physiopathology of Obesity and Nutrition), Instituto de Salud Carlos III, 28029 Madrid, Spain

**Keywords:** metabolic syndrome, gastrointestinal microbiome, probiotics, obesity

## Abstract

The aim of this systematic review is to evaluate whether the use of probiotics has any effect on the components of metabolic syndrome (MetS) before patients develop type 2 diabetes. A qualitative systematic review, following the Cochrane methodology, and a comprehensive literature search of randomized controlled trials (RCTs) were conducted in PubMed and Scopus from inception until 4 July 2019. According to our inclusion criteria, nine clinical studies were finally analyzed, corresponding to six RCTs. Probiotics intake in patients with MetS resulted in improvements in body mass index, blood pressure, glucose metabolism, and lipid profile in some studies. Regarding inflammatory biomarkers, probiotics also positively affected the soluble vascular cell adhesion molecule 1 (sVCAM-1), interleukine-6 (IL-6), tumor necrosis factor α (TNF-α), vascular endothelial growth factor (VEGF), and thrombomodulin. Despite the diversity of the published studies, the intake of probiotics for patients with MetS may offer a discrete improvement in some of the clinical characteristics of the MetS and a decrease in inflammatory biomarkers. Nevertheless, these beneficial effects seem to be marginal compared to drug therapy and a healthy lifestyle and clinically non-relevant.

## 1. Introduction

Obesity is increasing worldwide and is associated with the development of metabolic syndrome (MetS), which is a cluster of cardio-metabolic risk factors and comorbidities conveying high risk of both cardiovascular disease (CVD) and type 2 diabetes [1]. MetS is characterized by increased white adipose tissue and insulin resistance, which leads to an expanded risk of CVD [2]. According to the International Diabetes Federation (IDF), MetS is defined by the presence of central obesity plus any two of the following four factors: Raised triacylglycerols (>150 mg/dL) or specific treatment for this lipid abnormality; reduced high-density lipoprotein cholesterol (HDLc) (<40 mg/dL in males, <50 mg/dL in females) or specific treatment for this lipid abnormality; raised blood pressure (BP) (systolic BP > 130 mm Hg or diastolic BP > 85 mm Hg) or specific treatment for previously diagnosed hypertension; raised fasting plasma glucose (FPG > 100 mg/dL) or prediabetes [3].

In recent years, it has become apparent that the alteration of the gastrointestinal microbiota composition can contribute to the development of insulin resistance associated with obesity [4,5,6]. The gastrointestinal microbiota constitutes the set of microorganisms that reside in the gastrointestinal tract. The vast majority of microbes that exist in the human gastrointestinal tract live in the colon. More than 90% of all bacterial phylogenetic types belong to only two of the 70 divisions known (phyla) in the bacterial domain: Bacteroidetes and Firmicutes [7].

In this regard, a decreased ratio of Bacteroidetes/Firmicutes has been described in obese compared to lean subjects [8]. However, there is a lack of consistency among studies. It has been recently hypothesized that low microbial gene richness is a good marker for MetS [9]. Also, an aberrant gut microbiota can promote subacute systemic inflammation, resistance to insulin, and increased risk of CVD, due to mechanisms’ exposure to bacterial products, namely bacterial lipopolysaccharide (LPS) [10].

Furthermore, it has been proposed that probiotic intake may ameliorate some of the clinical components of MetS [11]. The term probiotic is currently used to describe bacteria associated with beneficial effects for humans and animals [FAO/WHO Expert Consultation 2001]. The most accepted definition of probiotics is “live microorganisms, which when consumed in adequate amounts, confer a health effect on the host” [12]. However, discrepant data regarding the health benefits of probiotics on metabolic diseases have been described. Some of the studies have found a beneficial effect in some of the components of MetS (BP and lipid profile), whereas contradictory results have been described on their effect on BMI in adult population [13]. These differences can be partially explained because of different study designs and the use of different probiotic strains, dose, and administration form.

For this reason, and to summarize the current status of the available evidence, our group has performed a systematic review of randomized controlled trials (RCTs) in MetS. This review aimed to answer the question: “Does the use of probiotics have any effect on the components of MetS as defined by the IDF before the development of type 2 diabetes?”

## 2. Materials and Methods

### 2.1. Search Strategy

We performed a qualitative systematic review of RCTs, published in English, conducted in adults with a diagnosis of MetS according to the IDF criteria. Pubmed (US National Library of Medicine National Institutes of Health), and Scopus databases and the Cochrane Collaboration were used for the literature search. The last day of publication included in this review was 4 July 2019. This literature review has been registered at the PROSPERO (International prospective register of systematic reviews) website on 8 September 2019, with the following record CRD42019142042. Available from: https://www.crd.york.ac.uk/prospero/display_record.php?ID=CRD42019142042.

For the Pubmed database, we used the following search equation: (“Obesity” [All fields] OR “overweight” [All fields] OR “Metabolic Syndrome” [All fields] OR “Abdominal obesity metabolic syndrome” [Supplementary Concept]) AND “Probiotics” [All fields]. As additional filters, we used Humans, Adults (+19 years old), and Randomized Controlled Trials published in English.

For the Scopus database, we identified the records by applying the following formula: TITLE-ABS-KEY ((“Obesity” OR “overweight” OR “Metabolic Syndrome” OR “Abdominal Obesity metabolic syndrome” AND “probiotics”)) AND ((clinical AND study)) AND ((“Single-Blind Method” OR “Cross-Over Studies” OR “Placebos” OR “multicenter study” OR “double blind procedure” OR “single blind procedure” OR “crossover procedure” OR “clinical trial” OR “controlled study” OR “randomization” OR “placebo”)) AND LIMIT-TO (SRCTYPE, “j”)) AND (LIMIT-TO (DOCTYPE, “ar”)) AND (LIMIT-TO (LANGUAGE, “English”)).

### 2.2. Data Handling, Analysis, Extraction, and Selection Criteria

Articles were selected based on the following inclusion criteria: (i) Intervention studies with probiotics compared to placebo with an RCT design, (ii) intervention studies in adults (age range 18–65) with a diagnosis of MetS according to the IDF criteria. Exclusion criteria were interventions with (i) synbiotics, (ii) prebiotics, (iii) without a proper placebo group, (iv) non-RCT design, (v) performed in children and adolescents, and (vi) articles focusing on people with type 2 diabetes, or pregnant and breastfeeding women. We decided to exclude individuals with type 2 diabetes since many of the specific treatments may have an effect on anthropometric parameters and cardiovascular risk, which are the primary outcomes of our review [14].

**Figure 1:**
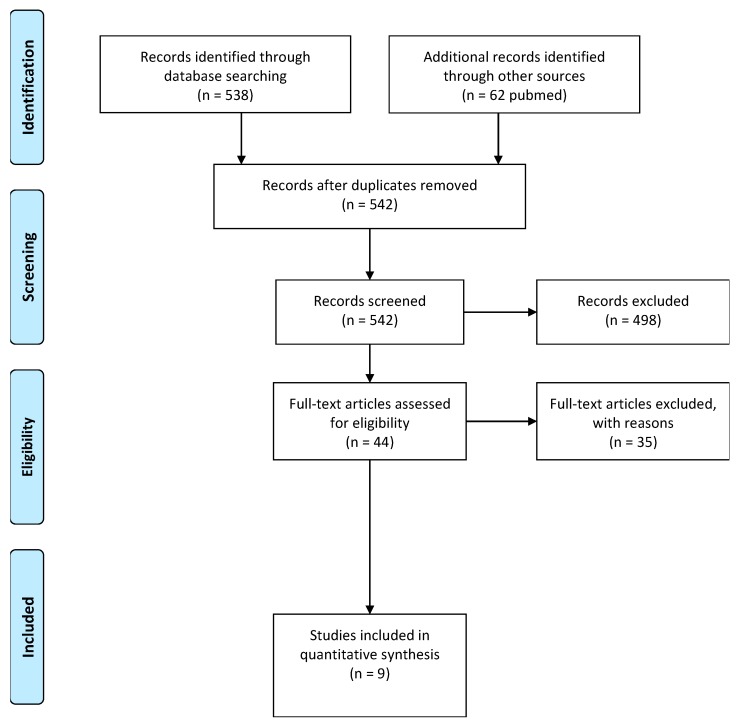
PRISMA flowchart of the literature search process.

The selection was performed by two authors (C.T.-J. and C.G.-L.), who worked independently based on PICO criteria (population, intervention, comparison, outcome) represented in Table 1. In case of discrepancies, a third independent reviewer (A.G.) was consulted for the final decision. Figure 1 represents the procedure that we used for the literature search. Articles were excluded based on the aforementioned exclusion criteria.

The primary outcomes were changes in any of the clinical parameters included in the definition of the MetS, which included BMI, waist circumference, glucose/HOMA index/insulin/glycated hemoglobin, BP, and lipid profile. Additional outcomes were changes in inflammatory parameters, gastrointestinal microbiota composition, and any related indirect measurement of the main components of the MetS and its relation to CVD.

### 2.3. Risk of Bias (Quality) Assessment

**Figure 2:**
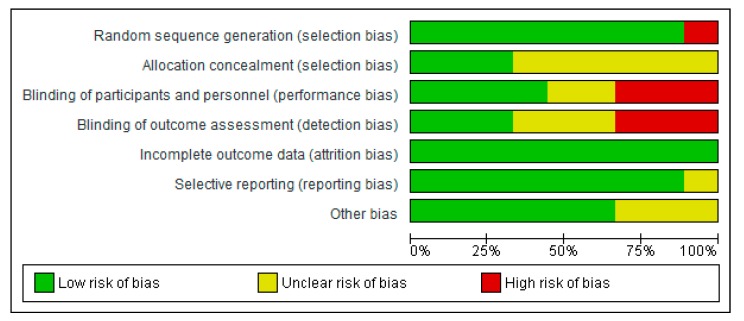
Risk of bias graph: Review authors’ judgments about each item presented as percentages across all included studies.

**Figure 3:**
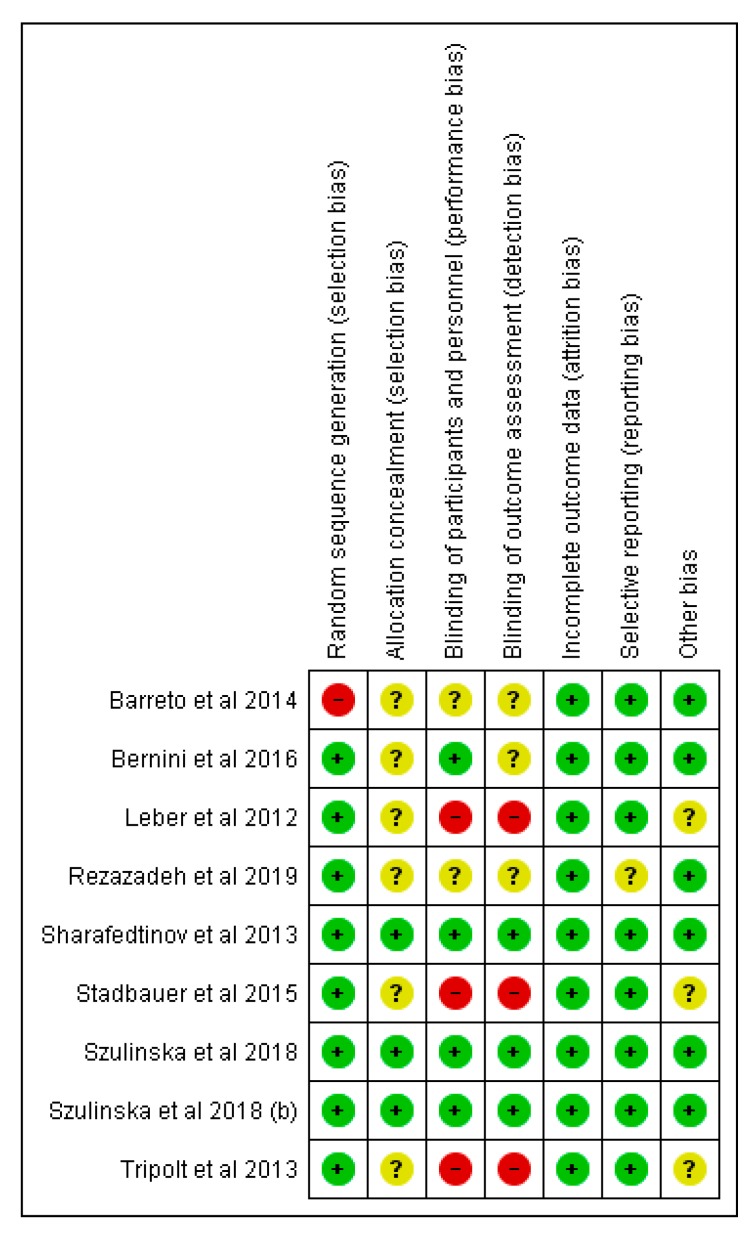
Risk of bias summary: Review authors’ judgments about each risk of bias item for each included study.

C.T.-J. and C.G.-L. independently assessed the risk of bias of the selected documents using the Cochrane collaboration’s methodology. In case of discrepancies, a third reviewer was involved in this evaluation (A.G.). Risk of bias was tabulated for each study and was classified as low, high, and unclear, as described in Chapter 8 of the Cochrane Handbook of Systematic Reviews of Interventions [15], and Figure 2; Figure 3 were generated in RevMan 5.3 (Copenhagen: The Nordic Cochrane Center, The Cochrane Collaboration, 2014).

### 2.4. Strategy for Data Synthesis

We provided a narrative synthesis of the main results in the selected trials, organized by specific clinical, biochemical, and gastrointestinal parameters. As a second level, we organized results by the type of probiotic strain.

## 3. Results

We found nine clinical studies in agreement with our inclusion criteria. The studies performed by Leber et al. [16], Tripolt et al. [17], and Stadlbauer et al. [18] accounted for the same clinical trial registration, as did the two studies conducted by Szulinska [19,20]. Six of them were performed in both men and women, whereas three of them were performed in postmenopausal women. The dose used and the duration of the intervention varied from 10^8^ cells/mL to 1.5 × 10^11^ colony forming units (CFU)/g, and from 3 to 12 weeks, respectively. The main results for primary and secondary outcomes, specific strains used, and characteristics of the studies are described in Table 2.

The probiotic strain was chosen in the different articles for their ability to improve gut permeability in rats [16]; their ability to reduce the gram-negative bacteria and increase bifidobacteria [17]; for their moderate anti-oxidative ability and their cholesterol-lowering efficacy in adults [21]; for their ability to lower plasma glucose, insulin, triacylglycerols, and oxidative stress [22]; for their ability to modulate natural killer cell and neutrophil function [18]; for their capacity to improve insulin resistance and reduce abdominal obesity in type 2 diabetes patients [19,20]; and there was not a stated reason in two studies [23,24].

We found that there was an improvement in some of the clinical components of the MetS in five out of the nine articles analyzed, specifically, BMI, glucose metabolism, and lipid profile [19,21,22,23,24]. Only one trial analyzed markers of endothelial function, showing a reduction of arterial stiffness related parameters, with no significant reductions in BP [20]. We also found an improvement in some of the inflammatory biomarkers; in particular, in the soluble vascular cell adhesion molecule 1 (sVCAM-1), interleukine-6 (IL-6), tumor necrosis factor α (TNF-α), vascular endothelial growth factor (VEGF), uric acid, homocysteine, LPS, and thrombomodulin as an endothelial cell damage marker [17,19,20,22,23,24]. Conversely, in the clinical trial NCT01182844, the ultrasensitive C-reactive protein (CRP), a well-known inflammatory biomarker, increased [16,17]. Regarding the effect of probiotics on the gastrointestinal microbiota composition, there are few data about it, although with non-specific changes reported [18,21].

### Assessment of Risk of Bias

All selected articles were assessed for risk of bias, in agreement with the Cochrane recommendations. Figure 2 and Figure 3 show the distribution of the risk of bias according to each category and domain specified in the Cochrane collaboration tool. Evaluation of the studies showed a low risk of bias in the following categories: Random sequencing generation (89%), incomplete outcome data (100%), and selective reporting (89%). Notwithstanding, allocation concealment category had a high percentage of unclear risk of bias (67%), whereas blinding of outcome assessment showed a 33% of low risk, unclear risk, and a high risk of bias. In the case of blinding of participants and personnel, we also found a 33% of high risk of bias.

The article of Sharafedtinov et al. and the two articles of Szulinska et al. were found to have the lowest risk of bias in the reporting of all items. Selection, performance, and detection bias was considered to have an unclear or high risk of bias in five out of the nine articles included. We believe that the authors should properly describe the type of blinding implemented or the impossibility of blinding.

## 4. Discussion

The main findings of the present review were that some of the selected probiotics are able to discretely improve some of the clinical components of the MetS, as well as some inflammatory biomarkers associated with the syndrome. Although significant, these supposedly positive effects are clinically non-relevant.

Regarding other similar studies, there is a previously published meta-analysis on probiotic foods and supplements interventions, including 18 RCTs with at least one of the criteria included in the definition of MetS [25]. They described a marginal and non-relevant effect on body fat percentage (−0.30%) and on low-density lipoprotein cholesterol (LDLc) levels (−0.16 mg/dL). Although we have not performed a meta-analysis, in both cases, an effect on some of the MetS components has been found. However, the validity of the latter meta-analysis for extracting meaningful information about the MetS is more than dubitable, due to the heterogeneity of the studied populations, namely: Pregnant women, children and adolescents, people with type 2 diabetes, high BP, or overweight.

In addition, there has been another published meta-analysis evaluating the effect of probiotics on overweight and obese subjects. The authors reported a decrease in BMI and body weight in the intervention group. Although they found some significant changes, they were clinically irrelevant (−0.6 kg, −0.27 kg/m^2^) [26].

Similarly, the effects of probiotic supplementation have been also analyzed in a meta-analysis of RCTs in patients with type 2 diabetes mellitus. They found a modest reduction in fasting glucose levels and an improvement in oxidative stress biomarkers [27].

Currently, to the best of our knowledge, there has not been a meta-analysis only in patients with a specific diagnosis of MetS published, excluding people with type 2 diabetes. MetS, as a cluster of anthropometric, clinicalm and metabolic abnormalities, has had different definitions developed under the auspices of diverse scientific societies and some of the cut-offs of its criteria vary [28]. In the present review, the included studies used the National Cholesterol Education Program Adult Treatment Panel III criteria [16,17,18,22,23], obesity accompanied by arterial hypertonia [21], and the IDF criteria [19,20,24].

To answer the question “Does the use of probiotics strains have any effect on the components of MetS, as defined by the IDF, before the development of type 2 diabetes?” a meta-analysis could not be performed, due to the design and methodology diversity and the small number of RCTs [29]. As opposed to the meta-analyses conducted in drugs, those conducted in nutritional science are not always the best method for extracting relevant information, due to the heterogeneity of interventions and protocols [29]. Indeed, one important issue that this review highlights is the heterogeneity of the studies, in terms of population, probiotic strain and genus, administered doses, and the period of the interventions.

Regarding the subjects included, some of the studies only recruited women, whereas the rest studied the probiotics in men and women in different proportions. From a clinical point of view, there may be a different cardiovascular risk in women than in men with MetS; however, the clinical implications of this issue are currently unknown [30].

Different strains have been used, and also some of the clinical studies used a mix of different probiotic strains. It is well-known that the probiotic effects are strongly strain-dependent. Type of strain used has mostly been selected due to previous beneficial effects in animals; however, animals’ results cannot always be transferred to human studies. Furthermore, the dose range, varying from 10^8^ to 10^11^ cells or CFU, and the administration form (cheese, yogurt, and milk or probiotic capsule), can also account for additional differences.

In addition, in three of the studies included, the time of exposure (3–6 weeks) could have not been long enough to demonstrate changes in some of the parameters related to glucose metabolism and insulin-resistance, such as glycated hemoglobin (HbA1c), the main diabetes control marker in clinical practice [31]. This is due to the fact that HbA1c depends on the lifespan of erythrocytes and reflects the glycemic control for the last two to three months [32].

On the other hand, only two of the nine reviewed articles studied the gut microbiota composition (Stadlbauer et al., Leber et al.). Nowadays, there is scientific evidence of the implication of the gastrointestinal microbiota in the development of MetS [4,5,6,7]. Therefore, the determination of the gastrointestinal composition should be implemented in RCTs with probiotics. In this sense, metagenomics can provide important insights into the functional ecology microorganisms.

Finally, one important limitation of this study is that we have only located nine articles meeting our criteria, which correspond to just six registered RCTs. However, we feel that our revision provides an updated picture of the available evidence.

## 5. Conclusions

According to the available data, most of the analyzed articles describe that the addition of probiotics to the treatment of patients with MetS may offer a discrete advantage to the current medical treatment in terms of improvement of some, but not all, of the clinical characteristics of the MetS and related inflammatory biomarkers. Although we cannot conclude that probiotics exert beneficial effects, they may have some positive effects that are marginal compared to drug therapy and a healthy lifestyle, and probably dose- and strain-specific. Also, the duration of the interventions may have not been long enough to prove the decrease in MetS associated complications.

For this reason, there is a need for better RCTs in humans with MetS able to fully elucidate if probiotics can be actually used as a coadjuvant therapy for this pathology. In this sense, we suggest following these recommendations when designing RCTs: (1) A crossover design is a more appropriate approach to determine the health benefits of clinical interventions than a parallel design. (2) RCTs should be designed with a more appropriate estimation of the size and statistical power, duration, type of strain, dose, and mode of delivery. (3) Finally, special caution should be taken in the interpretation of results so as to not confound statistical significance with biological relevance or strength of evidence.

## Figures and Tables

**Table 1:** PICO (population, intervention, comparison, outcome) criteria for inclusion of studies.

Parameter	Inclusion Criteria
Population	Adults (18–65 years old) with Metabolic Syndrome (MetS)
Intervention	Probiotic strains
Comparison	Probiotic strains vs. placebo
Outcome	Improvement on clinical components of the MetS
Setting	Randomized Clinical Trials

**Table 2:** Main characteristics of the nine included articles evaluating the effect of probiotics on metabolic syndrome parameters.

Author	*n* (Sample Size)	Age Range	Probiotic Strain	Period of Intervention (Weeks)	Probiotic Dose	Primary Outcomes	Secondary Outcomes
Leber et al. [16]	28	Control group: 54.5 ± 8.9 Probiotic group: 51.5 ± 11.4	*Lactobacillus casei* Shirota	12	milk (65 mL bottles × 3/day) 10^8^ cells/mL	No changes were found in BMI, BP, waist circumference, triacylglycerols, TC, and fasting glucose levels.	High-sensitive CRP (1.86 mg/L in the probiotic group vs. −1.60 mg/L in the placebo group, *p* = 0.016) and LBP levels (5827 ng/mL in the probiotic group vs. −1510 ng/mL in the placebo group, *p* = 0.023) increased within the probiotic group
Sharafedtinov et al. [21]	40	Control group: 51.7 ± 12.1 Probiotic group: 52 ± 10.9	*Lactobacillus plantarum* TENSIA	3	cheese (50 g/day) 1.5 × 10^11^ CFU/g	BMI was significantly reduced in the probiotic group. (BMI variation in probiotic group −2 vs. −1.6 kg/m^2^ in the placebo group, *p* = 0.031).	A positive association was detected between TENSIA colonization and the extent of change of morning diastolic BP (*r* = 0.617, *p* = 0.0248)
Tripolt et al. [17]	28	Control group: 55 ± 9 Probiotic group: 51 ± 11	*Lactobacillus casei* Shirota	12	milk (65 mL bottles × 3/day) 10^8^ cells/mL	No changes were found in BMI, fasting plasma glucose levels, and HOMA-IR index.	Probiotic supplementation resulted in a significant reduction in sVCAM-1 level (−195 ng/mL in the probiotic group vs. 30 ng/mL in the placebo group, *p* = 0.008) and a significant increase in high-sensitive CRP level (1.86 mg/L in the probiotic group vs. −1.60 mg/L in the placebo group, *p* = 0.002)
Barreto et al. [22]	24	Control group: 63 ± 7.6 Probiotic group: 62 ± 4.35	*Lactobacillus plantarum*	12	milk (80 mL bottles × 1/day) 10^7^ CFU/g	Glucose levels showed a significant reduction in the FM group compared with the NFM group (Glucose variation in FM −10.5 vs. −3 mg/dL in NFM group, *p* = 0.037).	Homocysteine levels showed a significant reduction in the FM group compared with the NFM group *p* = 0.019).
Stadlbauer et al. [18]	28	Control group: 55 ± 9 Probiotic group: 51 ± 11	*Lactobacillus casei* Shirota	12	milk (65 mL bottles × 3/day) 10^8^ cells/mL	No changes were found in BMI, BP, waist circumference, triacylglycerols, and TC blood levels.	LcS administration was associated with subtle microbiota changes at a genus level (enrichment of Parabacteroidetes)
Bernini et al. [23]	51	No data	*Bifidobacterium lactis* HN019	6	milk(80 mL bottle × 1/day) 3.4 × 10^8^ CFU/mL	Significant differences in BMI variation (Probiotic group −1.3 vs. −0.3 kg/m^2^ in control group^,^ *p* = 0.017); TC variation (probiotic group −15 vs. 6 mg/dL in control group, *p* = 0.09) and LDLc variation (probiotic group −17.5 vs. −2 mg/dL in control group, *p* = 0.08)	Significant decrease in TNFα and IL−6 (*p* < 0.05) in the probiotic group.
Szulinska et al. [19]	81	Control group: 58.72 ± 7.25 Low dose group: 56.38 ± 6.55 High dose group: 55.16 ± 6.87	*Bifidobacterium bifidum* W23, *Bifidobacterium lactis* W51, *Bifidobacterium lactis* W52, *Lactobacillus acidophilus* W37, *Lactobacillus brevis* W63, *Lactobacillus casei* W56, *Lactobacillus salivarius* W24, *Lactococcus lactis* W19, and *Lactococcus lactis* W58	12	lyophilisate powder Low dose (2.5 × 10^9^ CFU/day) or High dose (1 × 10^10^ CFU/day)	Significant differences were found in glucose variation (HD vs. placebo −0.61 mg/dL, *p* = 0.0272; HD vs. LD −0.72 mg/dL, *p* = 0.0043), Insulin (HD vs. placebo −0.83 UI/L, *p* = 0.0002; HD vs. LD −0.40 UI/L, *p* = 0.0155), and HOMA-IR (HD vs. placebo −0.90, *p* = 0.0005; HD vs. LD −0.54 mg/dL, *p* = 0.0127).	Significant differences were found in uric acid (HD vs. placebo −0.73 mmol/L, *p* = 0.0109; HD vs. LD −0.92 mmol/L, *p* = 0.0016) and LPS levels (HD vs. placebo −0.99 ng/mL, *p* = 0.001).
Szulinska et al. [20]	81	Control group: 58.72 ± 7.25 Low dose group: 56.38 ± 6.55 High dose group: 55.16 ± 6.87	*Bifidobacterium bifidum* W23, *Bifidobacterium lactis* W51, *Bifidobacterium lactis* W52, *Lactobacillus acidophilus* W37, *Lactobacillus brevis* W63, *Lactobacillus casei* W56, *Lactobacillus salivarius* W24, *Lactococcus lactis* W19, and *Lactococcus lactis* W58	12	lyophilisate powder Low dose (2.5 × 10^9^ CFU/day) or High dose (1 × 10^10^ CFU/day)	No changes were found in BMI and BP.	Significant differences were found in the pulse wave analysis systolic pressure (HD vs. placebo −1 mmHg, *p* = 0.0054; HD vs. LD −0.91 mmHg, *p* = 0.0057), the pulse wave analysis augmentation index (HD vs. placebo −0.55, *p* = 0.0079), the pulse wave velocity (HD vs. placebo −0.82 m/s, *p* = 0.0045; HD vs. LD −0.55 m/s, *p* = 0.0189), VEGF (HD vs. placebo −1.09 pg/mL, *p* = 0.0001; HD vs. LD −1.10 pg/mL, *p* = 0.0007), TNFα (HD vs. placebo −1.03 pg/mL, *p* = 0.0009; HD vs. LD −0.68 pg/mL, *p* = 0.0471), and thrombomodulin levels (HD vs. placebo −0.78 ng/mL, *p* = 0.0194).
Rezazadeh et al. [24]	44	Control group: 44.55 ± 5.70 Probiotic group: 44.05 ± 6.60	*Lactobacillus acidophilus* La5, *Bifidobacterium lactis* Bb12	8	yogurt containing 6.45 × 10^6^ CFU/g of *L. acidophilus* and 4.94 × 10^6^ CFU/g of *B. lactis* Bb12	Consumption of probiotic yogurt resulted in a significant reduction in the level of blood glucose (Mean difference: −3.80, *p* = 0.01)	Consumption of probiotic yogurt resulted in a significant reduction in the level of VCAM-1 (Mean difference −463.39, *p* = 0.001)

Abbreviations: SD: Standard deviation; BMI: Body mass index; BP: Blood pressure; TC: Total cholesterol; CRP: C reactive protein; LBP: Lipopolysaccharide binding protein; CFU: Colony forming units; VCAM-1: Vascular cell adhesion molecule 1; LDLc: Low-density lipoprotein cholesterol; FM: Fermented milk; NFM: Non-fermented milk; LcS: Lactobacillus casei Shirota; TNF-α: Tumor necrosis factor α; IL-6: Interleukine-6; HOMA-IR: Homeostasis model assessment-insulin resistance; LPS: Lipopolysaccharide; HD: High dose; LD: Low dose; VEGF: Vascular endothelial growth factor.

## References

[1] Zimmet P., Alberti K.G., Stern N., Bilu C., El-Osta A., Einat H., Kronfeld-Schor N. (2019). The Circadian Syndrome: Is the Metabolic Syndrome and much more!. J. Intern. Med..

[2] Alberti K.G., Eckel R.H., Grundy S.M., Zimmet P.Z., Cleeman J.I., Donato K.A., International Diabetes Federation Task Force on Epidemiology and Prevention, Hational Heart, Lung, and Blood Institute, American Heart Association, World Heart Federation (2009). Harmonizing the metabolic syndrome: A joint interim statement of the International Diabetes Federation Task Force on Epidemiology and Prevention; National Heart, Lung, and Blood Institute. Circulation.

[3] Alberti K.G., Zimmet P., Shaw J. (2006). Metabolic syndrome—A new world-wide definition. A consensus statement from the international diabetes federation. Diabet. Med..

[4] Bäckhed F., Ding H., Wang T., Hooper L.V., Koh G.Y., Nagy A., Semenkovich C.F., Gordon J.I. (2004). The gut microbiota as an environmental factor that regulates fat storage. Proc. Natl. Acad. Sci. USA.

[5] Bäckhed F., Manchester J.K., Semenkovich C.F., Gordon J.I. (2007). Mechanisms underlying the resistance to diet-induced obesity in germ-free mice. Proc. Natl. Acad. Sci. USA.

[6] Turnbaugh P.J., Ley R.E., Mahowald M.A., Magrini V., Mardis E.R., Gordon J.I. (2006). An obesity-associated gut microbiome with increased capacity for energy harvest. Nature.

[7] Turnbaugh P.J., Ley R.E., Hamady M., Fraser-Liggett C.M., Knight R., Gordon J.I. (2007). The human microbiome project. Nature.

[8] Turnbaugh P.J., Hamady M., Yatsunenko T., Cantarel B.L., Duncan A., Ley R.E., Sogin M.L., Jones W.J., Roe B.A., Affourtit J.P. (2009). A core gut microbiome in obese and lean twins. Nature.

[9] Le Chatelier E., Nielsen T., Qin J., Prifti E., Hildebrand F., Falony G., Almeida M., Arumugam M., Batto J.M., Kennedy S. (2013). Richness of human gut microbiome correlates with metabolic markers. Nature.

[10] Plovier H., Cani P.D. (2017). Microbial Impact on Host Metabolism: Opportunities for Novel Treatments of Nutritional Disorders?. Microbiol. Spectr..

[11] Sáez-Lara M.J., Robles-Sanchez C., Ruiz-Ojeda F.J., Plaza-Diaz J., Gil A. (2016). Effects of Probiotics and Synbiotics on Obesity, Insulin Resistance Syndrome, Type 2 Diabetes and Non-Alcoholic Fatty Liver Disease: A Review of Human Clinical Trials. Int. J. Mol. Sci..

[12] Sanders M. (2008). Probiotics: Definition, sources, selection, and uses. Clin. Infact. Dis..

[13] Rondanelli M., Faliva M.A., Perna S., Giacosa A., Peroni G., Castellazzi A.M. (2017). Using probiotics in clinical practice: Where are we now? A review of existing meta-analyses. Gut. Microbes.

[14] Adeshirlarijaney A., Zou J., Tran H., Chassaing B., Gewirtz A.T. (2019). Amelioration of metabolic syndrome by metformin associates with reduced indices of low-grade inflammation independently of the gut microbiota. Am. J. Physiol. Endocrinol. Metab..

[15] Higgins J.P.T., Altman D.G., Sterne J.A.C., Higgins J.P.T., Green S. (2011). Chapter 8. Cochrane Handbook for Systematic Reviews of Interventions, Version 5.1.0.

[16] Leber B., Tripolt N.J., Blattl D., Eder M., Wascher T.C., Pieber T.R., Stauber R., Sourij H., Oettl K., Stadlbauer V. (2012). The influence of probiotic supplementation on gut permeability in patients with metabolic syndrome: An open label, randomized pilot study. Eur. J. Clin. Nutr..

[17] Tripolt N.J., Leber B., Blattl D., Eder M., Wonisch W., Scharnagl H., Stojakovic T., Obermayer-Pietsch B., Wascher T.C., Pieber T.R. (2013). Short communication: Effect of supplementation with Lactobacillus casei Shirota on insulin sensitivity, β-cell function, and markers of endothelial function and inflammation in subjects with metabolic syndrome—A pilot study. J. Dairy Sci..

[18] Stadlbauer V., Leber B., Lemesch S., Trajanoski S., Bashir M., Horvath A., Tawdrous M., Stojakovic T., Fauler G., Fickert P. (2015). Lactobacillus casei Shirota supplementation does not restore gut microbiota composition and gut barrier in metabolic syndrome: A randomized pilot study. PLoS ONE.

[19] Szulińska M., Łoniewski I., van Hemert S., Sobieska M., Bogdański P. (2018). Dose-Dependent Effects of Multispecies Probiotic Supplementation on the Lipopolysaccharide (LPS) Level and Cardiometabolic Profile in Obese Postmenopausal Women: A 12-Week Randomized Clinical Trial. Nutrients.

[20] Szulińska M., Łoniewski I., Skrypnik K., Sobieska M., Korybalska K., Suliburska J., Bogdański P. (2018). Multispecies Probiotic Supplementation Favorably Affects Vascular Function and Reduces Arterial Stiffness in Obese Postmenopausal Women-A 12-Week Placebo-Controlled and Randomized Clinical Study. Nutrients.

[21] Sharafedtinov K.K., Plotnikova O.A., Alexeeva R.I., Sentsova T.B., Songisepp E., Stsepetova J., Smidt I., Mikelsaar M. (2013). Hypocaloric diet supplemented with probiotic cheese improves body mass index and blood pressure indices of obese hypertensive patients—A randomized double-blind placebo-controlled pilot study. Nutr. J..

[22] Barreto F.M., Colado Simão A.N., Morimoto H.K., Batisti Lozovoy M.A., Dichi I., Helena da Silva Miglioranza L. (2014). Beneficial effects of Lactobacillus plantarum on glycemia and homocysteine levels in postmenopausal women with metabolic syndrome. Nutrition.

[23] Bernini L.J., Simão A.N., Alfieri D.F., Lozovoy M.A., Mari N.L., de Souza C.H., Dichi I., Costa G.N. (2016). Beneficial effects of Bifidobacterium lactis on lipid profile and cytokines in patients with metabolic syndrome: A randomized trial. Effects of probiotics on metabolic syndrome. Nutrition.

[24] Rezazadeh L., Gargari B.P., Jafarabadi M.A., Alipour B. (2019). Effects of probiotic yogurt on glycemic indexes and endothelial dysfunction markers in patients with metabolic syndrome. Nutrition.

[25] Dong Y., Xu M., Chen L., Bhochhibhoya A. (2019). Probiotic Foods and Supplements Interventions for Metabolic Syndromes: A Systematic Review and Meta-Analysis of Recent Clinical Trials. Ann. Nutr. Metab..

[26] Borgeraas H., Johnson L.K., Skattebu J., Hertel J.K., Hjelmesaeth J. (2018). Effects of probiotics on body weight, body mass index, fat mass and fat percentage in subjects with overweight or obesity: A systematic review and meta-analysis of randomized controlled trials. Obes. Rev..

[27] Ardeshirlarijani E., Tabatabaei-Malazy O., Mohseni S., Qorbani M., Larijani B., Baradar Jalili R. (2019). Effect of probiotics supplementation on glucose and oxidative stress in type 2 diabetes mellitus: A meta-analysis of randomized trials. DARU.

[28] Dabke K., Hendrick G., Devkota S. (2019). The gut microbiome and metabolic syndrome. J. Clin. Investig..

[29] Barnard N.D., Willett W.C., Ding E.L. (2017). The Misuse of Meta-analysis in Nutrition Research. JAMA.

[30] Rochlani Y., Pothineni N.V., Mehta J.L. (2015). Metabolic Syndrome: Does It Differ between Women and Men?. Cardiovasc. Drugs Ther..

[31] American Diabetes Association (2019). 6. Glycemic Targets: Standards of Medical Care in Diabetes-2019. Diabetes Care.

[32] Kojić Damjanov S., Đerić M., Eremić Kojić N. (2014). Glycated hemoglobin A1c as a modern biochemical marker of glucose regulation. Med. Pregl..

